# Enhanced rubber yield and its stability during the rainy season: insights from five-year yield monitoring in Southwestern China

**DOI:** 10.3389/fpls.2026.1824436

**Published:** 2026-05-01

**Authors:** Ran Wei, Chunguang Suo, Jiang Xie, Qiliang Yang, Die Feng, Yiling Feng, Jixiang Li, Yue Li

**Affiliations:** 1 Faculty of Science, Kunming University of Science and Technology, Kunming, China; 2 Yunnan Institute of Tropical Crops, Jinghong, Yunnan, China; 3 Faculty of Modern Agricultural Engineering, Kunming University of Science and Technology, Kunming, China

**Keywords:** climate change, rubber yield, soil carbon, soil chemical properties, yield stability

## Abstract

**Introduction:**

To meet the growing challenges for sustainable rubber production and rising market demand, rubber plantations should aim to optimize yield and maintain higher yield stability despite seasonal environmental fluctuations. However, the effects of rainy and dry seasons on rubber yield, yield stability, and soil properties as well as the potential links between them remain poorly understood.

**Methods:**

We conducted a five-year field experiment to examine the influence of rainy and dry seasons on rubber yield, yield stability, and soil properties. Rubber yield was recorded annually from 2014 to 2018, while soil properties were measured during one seasonal cycle (2017–2018) at the endpoint of the five-year experiment. Linear mixed-effects models were used for statistical analysis.

**Results:**

Across the five years, rubber yield during the rainy season was 52.3% higher than in the dry season, and the yield stability index was 34.6% higher. Linear mixed-effects models showed that the seasonal effect on yield did not reach statistical significance (p = 0.054), while the effect on stability was marginally significant (*p* = 0.049). Soil carbon (C) and nitrogen (N) contents showed no significant changes between seasons (*p* > 0.05 for both). No clear correlation was found between rubber yield or yield stability and soil C and N changes.

**Discussion:**

Our results suggest a numerical trend toward higher rubber yield and stability in the rainy season, which may be partly attributed to elevated precipitation and temperature. Longer-term continuous observations (≥ 10 years) are warranted to validate these findings and to clarify the role of soil chemical processes in driving plantation productivity.

## 1 Introduction

In recent years, the global demand for natural rubber has risen substantially (Fox et al., 2014). This growing demand has driven the expansion of rubber plantations into regions characterized by distinct rainy and dry seasons, thereby amplifying climate-related challenges (Fox and Castella, 2013). Thus, achieving smart and sustainable agriculture with respect to climate change impacts and complexities remains a key global challenge (Agovino et al., 2019; Walter et al., 2017). Additionally, seasonal leaf growth significantly affects resource assimilation and economic returns in rubber plantations under varying water conditions (Cotter et al., 2017). Therefore, investigating the long-term seasonal dynamics of rubber tree growth and latex production will provide key information to develop strategies to address future climate changes. Currently, most research focuses on rubber production or absolute changes under a single, specific climate condition (e.g., constant temperature and precipitation without seasonal variation) (Zomer et al., 2014). However, the growth response and latex production of rubber trees under different rainy and dry season conditions have not been fully explored.

Amid intensifying climate change and agricultural intensification, interannual yield stability has emerged as a pivotal concern (Knapp and van der Heijden, 2018). Yield stability, quantified as the degree of consistency in interannual latex production (i.e., low interannual variability), serves as a critical indicator of plantation resilience (Ray et al., 2015). High yield stability is essential for maintaining consistent management stable economic returns in rubber plantations under escalating environmental fluctuations (Chen et al., 2022a), and it directly impacts long-term rubber supply (Schrama et al., 2018). Studies suggest that yield stability, rather than peak latex production, may play a more decisive role in meeting global demand for agricultural commodities like rubber under fluctuating climate conditions (Raseduzzaman and Jensen, 2017). Building on this premise, researchers hypothesize that enhancing soil health (e.g., maintaining soil organic matter and nutrient content) could stabilize yields (King and Blesh, 2018; McDaniel et al., 2014). However, few studies have systematically examined the effects of climate variability on rubber yield stability over an extended period, limiting the ability to draw robust conclusions.

Soil carbon (C) and nitrogen (N) stocks reflect the balance between inputs (e.g., organic matter inputs from litter decomposition, root exudation, and manure application) and outputs (e.g., gaseous losses via soil respiration and denitrification, leaching of dissolved organic C/N, and biomass removal through harvesting) (Cleveland et al., 2006). In rubber plantation systems, changes in soil C and N content may decouple from total biomass removal due to variations in soil carbon stability or belowground biomass production (Monforti et al., 2015; Sanderman et al., 2017). Agricultural ecosystems, comprising abundant soil C and N reservoirs, play a critical role in climate change mitigation (Davis et al., 2016). Even a marginal reduction in soil C stock can substantially elevate atmospheric CO_2_ concentrations (Schmidt et al., 2011), while soil N dynamics may cascade into N_2_O emissions, nitrate leaching, altered nutrient availability, and ultimately affect agricultural sustainability (Omonode and Vyn, 2006). Together, these factors pose a substantial threat to the resilience of agroecosystems (Müller et al., 2011). Moreover, soil phosphorus (P) and potassium (K) are important nutrient elements, and any fluctuation in their content is a key factor for evaluating rubber yield and guiding soil fertilization strategies (Kamran et al., 2023). Yet, the interplay between rubber yield; soil C, N, K, and P stocks; and climatic drivers—such as nutrient availability (Zhang et al., 2015), climate variables (Myers et al., 2017), and management practices (Davis et al., 2016) remains underexplored. This limits the understanding, application of relevant strategies, and improvement of yield and yield stability on a broader scale.

In this study, we simultaneously investigated rubber yield, yield stability, and soil chemical properties (including soil organic carbon content, soil total nitrogen content, soil available phosphorus content, and soil available potassium content) from a 5-year extended period in Southwestern China. We hypothesized that elevated precipitation and temperature in the rainy season would increase rubber yield and yield stability. Regarding soil chemical properties, we tested the null hypothesis that soil organic carbon (C) and total nitrogen (N) contents remain stable across rainy and dry seasons due to the balanced C/N input-output dynamics typical of tropical red soil in rubber plantations. For soil available phosphorus (P) and potassium (K), we hypothesized that they would decrease during the rainy season due to increased plant uptake and leaching. Our objectives were to explore the impacts of rainy season and dry season on (1) rubber yield and its stability and (2) changes in soil chemical properties in different soil layers across the five years.

## 2 Materials and methods

### 2.1 Study site

**Figure 1:**
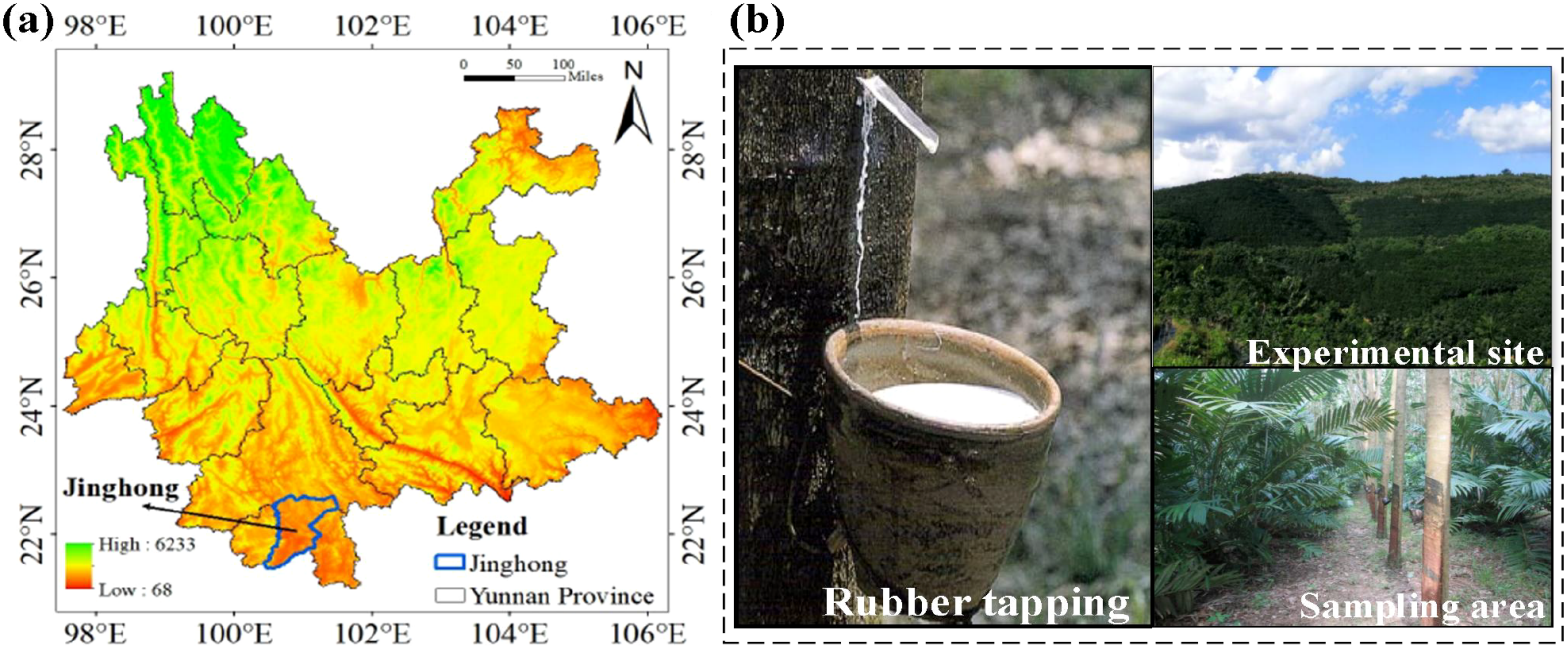
**(a)** The location of experimental site and **(b)** representative sample of rubber tree tapping in experimental site.

**Figure 2:**
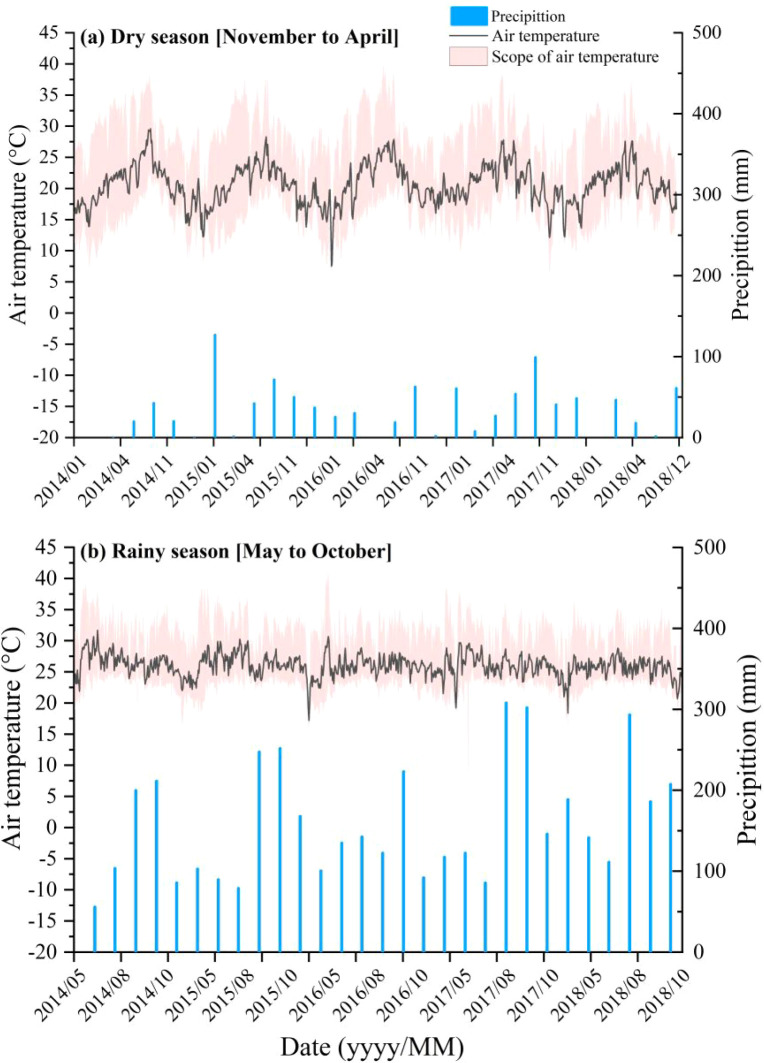
Daily precipitation (blue bars), daily average air temperature (black curves), and air temperature (light red ribbons) during **(a)** dry [November to April] and **(b)** rainy [May to October] seasons in Jinghong, Yunnan, China.

The study was conducted at the Yunnan Tropical Crop Science Research Institute in Jinghong, Yunnan, China (22°40’N, 101°50’E; 553 m a.s.l.; **Figure 1**). The site experiences a typical Southeast Asian tropical monsoon climate, characterized by distinct dry (November to April) and rainy (May to October) seasons. The mean annual temperature was 21.5 °C, the mean annual precipitation was 1,162 mm, and the evaporation was 1,311 mm (**Figure 2**, **Supplementary Figure 1**). The soil is classified as a Typic Latosol with a loamy-sandy texture, an average pH of 4.3 and a bulk density of 1.1 g cm^–3^. The area has a long history of cultivating tropical crops such as natural rubber. Further site details are available in Xiao et al. (2014) and Zhou et al. (2015).

### 2.2 Experimental design

A long-term field experiment was established in 2014 across five replicate blocks, covering a total area of approximately 1.2 ha. The plantations comprised at least 15-year-old rubber trees (*cv.* Yunyan 77-2), which were initially planted using a conventional method with a row spacing of 3 m × 8 m. Base fertilizers were applied consisting of 5 t ha^–1^ of farmyard manure (primarily cow dung) to improve soil organic matter, 0.8 t ha^–1^ of compound fertilizer (N:P:K = 15:10:12) to supply balanced primary nutrients, and 1 t ha^–1^ of calcium magnesium phosphate to provide phosphorus and ameliorate soil acidity.

### 2.3 Soil sampling and analysis

Soil samples were collected during one full seasonal cycle (dry season: November 2017 to April 2018; rainy season: May to October 2018) after five years of rubber yield monitoring. The objective was to compare soil chemical properties between the two precipitation seasons at the endpoint of the five-year experiment, rather than to track interannual soil dynamics. Soil samples were collected from the soil surface to 40 cm depth at typical locations within each sample plot. Twenty-four composite soil samples were collected across two seasons (dry and rainy) with three replicates per season and four sampling locations per replicate. Each composite soil sample contained 20 mixed soil cores, using a 5 cm diameter soil auger (5 cm diameter × 20 cm depth). The soil samples were air dried at 18–25°C for 14 days and sieved through a 1 mm sieve. Using sulfuric acid-potassium dichromate oxidation method, a wet oxidation technique, soil organic carbon concentration was determined in triplicates (Zhou et al., 2023). Soil total nitrogen concentration was determined using the semi-micro Kjeldahl method (Quirino et al., 2023). Available soil P content was measured using the Olsen method (Kleinman et al., 2009), while available soil K content was measured using flame photometry (Bar-Yosef and Akiri, 1978). The soil pH value was measured using a potentiometer with a 1:2.5 soil-to-water ratio.

### 2.4 Rubber yield and yield stability

Rubber yield was recorded after rubber tapping. Rubber trees were tapped once a month, and tapping was stopped in January and February each year to protect the trees (Xiao et al., 2014). To avoid boundary effects, the total rubber yield was calculated from the central 12 m × 32 m area of each plot. Yield stability (*S_i_*) was quantified for various planting systems (Tollenaar and Lee, 2002; Tian et al., 2016), and calculated based on the reciprocal of the coefficient of variation (Equation 1) (Li et al., 2020, Li et al., 2022; Chen et al., 2022a):

(1) $$S_{i}=\frac{Mean_{i}}{SD_{i}}$$

where *Mean_i_* and *SD_i_* refer to the mean and standard deviation of rubber yield in each treatment (i) over five rubber tree growing seasons. The stability index also referred to as the reciprocal of the coefficient of variation (*CV*), is a widely used metric in crop yield stability analysis (Equation 2):

(2) $$CV=\frac{SD_{i}}{Mean_{i}}\times 100\%$$

A higher *S_i_* value indicates greater yield stability (i.e., lower relative interannual variability). Since *S_i_* is mathematically equivalent to 1/*CV*, it is inherently influenced by changes in mean yield. To address this potential concern, we also calculated the coefficient of variation as a complementary measure of relative yield variability. Lower *CV* indicates higher stability. Both metrics are reported to provide a more robust assessment of seasonal differences in yield stability.

### 2.5 Statistical analysis

All statistical analyses were completed in R 4.0.2. All graphics were drawn using the ‘*ggplot2*’ package in R software. All data were tested for normal distribution using the Shapiro-Wilk method, and the Levene test was used to assess the equality of variance at *p* < 0.05. The effects of the rainy season on rubber yield, rubber yield, soil organic carbon content, soil total nitrogen content, soil available P, and soil available K were examined using a linear mixed effects model by ‘*nlme*’ package in R software. Regarding studying yield stability for 5 years, the precipitation season was set as a fixed effect, while plots nested within blocks were set as random effects. Additionally, to assess whether treatment differences were associated with soil organic carbon content, soil total nitrogen content, soil available P, soil available K content, precipitation season, growth year, and their interactions were set as fixed effects, while blocks were treated as random factors. Regarding the stability of rubber production, seasonal effects were set as fixed effects, while block effects were set as random effects. For each analysis, the effect was considered statistically significant when *p* < 0.05. Effects with *p* < 0.10 were considered marginally significant and interpreted as statistical trends requiring cautious interpretation. All residual variances and residuals met statistical requirements. The homogeneity of residual variance and the normality of residuals were also tested. Given that soil properties were measured during only one seasonal cycle (2017–2018) within the five-year yield monitoring period, linear mixed-effects models were used to account for repeated yield measurements; more complex approaches such as path analysis were not feasible due to insufficient temporal replication of soil data. Differences in coefficient of variation (*CV*) between seasons were compared using a paired t-test on the *CV* values calculated for each replicate across the five years. The correlation heatmap was generated using the ‘corrplot’ package in R, with hierarchical clustering based on Euclidean distance and Ward’s linkage method to group variables with similar correlation patterns.

## 3 Results and discussion

### 3.1 Rubber yield and yield stability across different precipitation seasons

**Figure 3:**
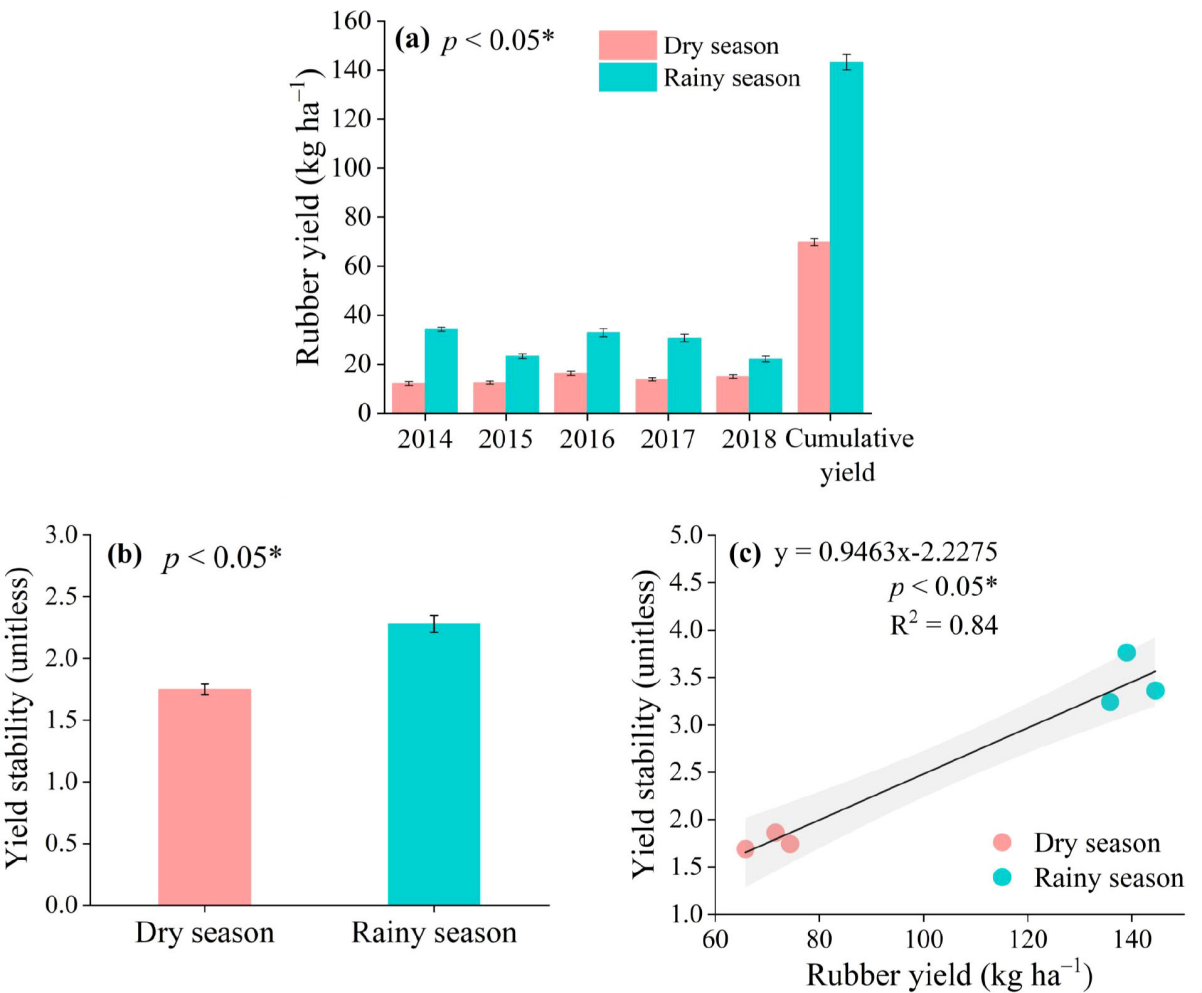
**(a)** Averaged rubber yield, **(b)** yield stability, and **(c)** relationship between rubber yield and yield stability during 2014–2018. The grey shaded area represents the 95% confidence intervals for the regression. Values represent mean ± standard errors (*n* = 3). Significant differences were evaluated at *p<0.05.

Across the five years, the cumulative rubber yield during the rainy season increased by 52.3% compared with the dry season (**Figure 3**). Specifically, compared with the dry season, the rubber yield increased in the rainy season by 64.4% in 2015, 46.5% in 2016, 50.1% in 2017, and 32.7% in 2018 (**Table 1**).To further evaluate whether the observed difference in *S_i_* is driven solely by mean yield differences, we compared the coefficient of variation (*CV*) between seasons. The *CV* for rubber yield was 0.23 ± 0.04 in the rainy season versus 0.31 ± 0.05 in the dry season, indicating lower relative interannual variability during the rainy season. Although the difference in *CV* did not reach statistical significance (*p* = 0.12), the direction of the effect is consistent with the *S_i_* results, supporting the interpretation that rainy season conditions are associated with numerically higher yield stability.

**Table 1:** Linear mixed-effects models of different seasons (S, dry and rainy seasons), growing years (Y), and their interactive effects on rubber yield and yield stability.

Data	Factors	numDF	denDF	*F* value	*P* value
Rubber yield	Intercept	1	19	407,654.76	< 0.05
	S	1	19	213.04	0.054
	Y	4	19	2898.23	< 0.05
	S×Y	4	19	13.22	0.082
Yield stability	Intercept	1	4	81,530.94	< 0.05
	S	1	4	42.61	0.049
	Rubber yield from each growing year				
2014	Intercept	1	4	231.85	< 0.05
	S	1	4	60.60	0.063
2015	Intercept	1	4	178.74	< 0.05
	S	1	4	62.37	0.128
2016	Intercept	1	4	245.95	< 0.05
	S	1	4	81.65	0.082
2017	Intercept	1	4	330.25	< 0.05
	S	1	4	69.32	0.074
2018	Intercept	1	4	185.83	< 0.05
	S	1	4	75.15	0.059

numDF, numerator degrees of freedom, denDF, denominator degrees of freedom. Linear mixed-effects models were performed when all growing year measurements are put together or separately for each growing year. For all observations, different seasons (S), growing year (Y) and their interactions (S × Y) were set as fixed factors, and plot was set as random factors, For observations from each growing year, S was set as a fixed factor, and plot was set as random factors. Dry season, November to April and rainy season, May to October.

The linear mixed-effects model showed that the seasonal effect on rubber yield had a p-value of 0.054 (**Table 1**). According to the threshold defined in Section 2.5, this result is considered marginally significant, indicating a statistical trend rather than a significant difference at α = 0.05. Similarly, the seasonal effect on yield stability had a p-value of 0.049, which reaches statistical significance. The marginal trend for yield (*p* = 0.054) carries ecological relevance: the 52.3% numerical increase in rainy season yield, though not statistically robust over the 5-year period, suggests that the physiological responses of rubber trees—such as enhanced photosynthetic activity, reduced water stress, and increased latex flow under warmer and wetter conditions—may cumulatively favor higher productivity. The lack of full statistical significance may stem from high interannual variability and the relatively short observation period, which limits the power to detect seasonal effects.

Notwithstanding the marginal statistical significance, the numerical increase (52.3%) in rainy season yield aligns with the physiological expectation that rubber trees benefit from ample precipitation and warm temperatures. Thus, ensuring sufficient precipitation and maintaining a high-temperature and high-humidity environment are of great importance for improving rubber yield and its stability, though longer-term monitoring is needed to confirm the statistical robustness of this trend. Abundant water conditions are critical for rubber trees to grow normally and produce rubber. An annual precipitation of 1,500 mm or more is reported to be adequate (Noguchi et al, 2003). Several other studies have shown that during periods of drought or seasonal dry conditions, rubber tree growth is inhibited, and leaf extraction slows, leading to shorter tapping durations and reduced rubber yield (Anna et al., 2020). However, excessive precipitation in form of heavy rains can elevate the risk of rubber diseases, potentially causing rubber washing and reducing overall production (Ahrends et al., 2015). Furthermore, interannual climate change is a possible explanation for the changes in rubber yield over the years (Ceglar et al., 2016; Ray et al., 2015). The research findings emphasize the importance of evaluating and understanding the impact of climate change on rubber yield and its stability, which may help strategize adaptation to climate change, optimize rubber planting, and improve yield.

Rubber trees possess unique physiological characteristics that influence seasonal yield dynamics. Unlike annual crops, rubber trees undergo a distinct phenological cycle: they shed leaves during the dry season and refoliate at the onset of the rainy season, with latex flow resuming after new leaves mature (Liu et al., 2025). During the rainy season, enhanced soil moisture and temperature promote metabolic activity, increase turgor pressure in laticifers, and reduce the incidence of tapping panel dryness, collectively facilitating higher latex yield (Noguchi et al., 2003). Conversely, water deficit during the dry season induces stomatal closure and restricts photosynthetic carbon assimilation, leading to reduced latex production. Existing research indicates that a mismatch between crop water demand and precipitation patterns during distinct rainy and dry seasons can constrain yield and its stability (Mo et al., 2009). Therefore, the higher yield observed during the rainy season in this study is consistent with rubber trees’ physiological adaptation to seasonal water availability.

### 3.2 Soil carbon, nitrogen, and other element content

**Figure 4:**
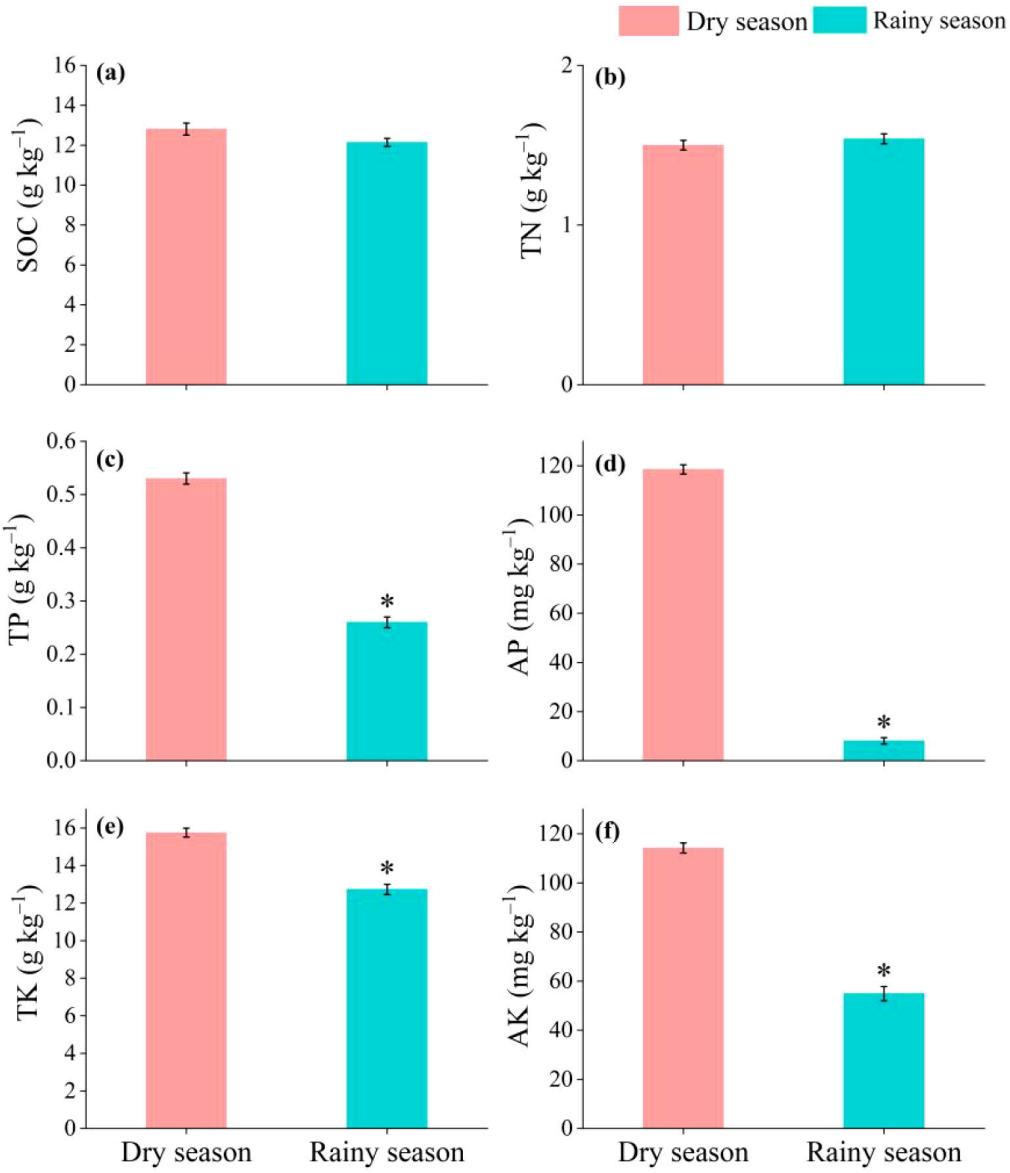
**(a)** Soil organic carbon (SOC), **(b)** soil total nitrogen (TN), **(c)** soil total phosphorus (TP), **(d)** soil available phosphorus (AP), **(e)** soil total potassium (TK), and **(f)** soil available potassium (AK) at 0–40 cm soil layers in different seasons (dry and rainy) in 2018. Values represent mean ± standard errors for three replicates (*n* = 3). Significant differences were evaluated at *p < 0.05.

The contents of carbon, nitrogen, phosphorus, and potassium in the soil from the surface to a depth of 40 cm (**Figure 4**) were measured. The results showed that in the soil from the surface to a depth of 40 cm, the effect of the rainy season (*vs.* dry season) on soil C and N content was not significant. In the linear mixed effects model, no interactions between the rainy season (*vs.* dry season) and year (**Table 2**) were observed. Contrary to our initial expectation, soil organic carbon (C) and total nitrogen (N) contents did not show significant changes between rainy and dry seasons. This finding supports the null hypothesis that the C and N input-output balance in tropical red soil under rubber plantations remains stable across seasons, and the rainy season does not substantially alter soil C and N content. We propose three mechanistic explanations grounded in the specific conditions of our study area: (i) Litter input from rubber trees increases during the rainy season due to higher leaf turnover and fine root mortality. However, warmer and wetter conditions also accelerate microbial decomposition, resulting in a compensatory balance between litter addition and mineralization. This dynamic equilibrium prevents net accumulation or loss of soil C and N over a single seasonal cycle (Fisk and Fahey, 2001; Ramirez et al., 2010; Vadeboncoeur, 2010). (ii) The Typic Latosol at our site has a high clay content (approximately 44%) and exhibits strong organic matter adsorption capacity, which inhibits the leaching of dissolved organic C and N even during high-intensity rainfall events. This buffering capacity maintains soil C and N pools despite seasonal fluctuations in water flux (Yang Y. et al., 2019). (iii) Rubber trees maintain a deep and extensive root system. Continuous root exudation and fine root turnover provide a steady supply of C and N to the soil throughout the year, independent of aboveground seasonal changes. This belowground input acts as a buffer, offsetting any seasonal surface imbalances. Taken together, these mechanisms explain why a 5-year observation period, with soil sampling conducted during one seasonal cycle, did not detect significant changes in soil C and N. Longer-term monitoring (≥ 10 years) may be required to capture potential slow trends in soil organic matter dynamics.

**Table 2:** Linear mixed-effects models of the effects of different seasons (S, dry and rainy seasons) on top layer (0–40 cm) soil organic carbon (SOC), soil total nitrogen (TN), soil total phosphorus (TP), soil available phosphorus (AP), soil total potassium (TK), and soil available potassium (AK).

Data	Factors	numDF	denDF	*F* value	*P* value
SOC	Intercept	1	19	6229.455	< 0.05
	S	1	19	1.058	0.732
TN	Intercept	1	19	4628.191	< 0.05
	S	1	19	0.977	0.864
TP	Intercept	1	19	353.112	< 0.05
	S	1	19	0.142	< 0.05
AP	Intercept	1	19	1411.796	< 0.05
	S	1	19	0.468	< 0.05
TK	Intercept	1	19	436.405	< 0.05
	S	1	19	0.873	< 0.05
AK	Intercept	1	19	1605.042	< 0.05
	S	1	19	0.481	< 0.05

numDF, numerator degrees of freedom, denDF, denominator degrees of freedom. Linear mixed-effects models were conducted, seasons (S) was considered as fixed factors, while plot within block were considered as random factor. Dry season, November to April and rainy season, May to October.

It should also be noted that observations of soil C and N content require further research on the input and output balance, which thus, entailing a longer duration to observe changes in their patterns. Thus, a 5-year period may be insufficient to detect significant changes (Congreves et al., 2017). Factors significantly affecting soil C and N contents must be continuously observed in prolonged planting systems (West and Post, 2002). Moreover, continuous tapping and collection of rubber at the research site might have reduced some of the plant derived C and N inputs, which may have restricted the storage of soil C and N (Chen et al., 2022b; Wang et al., 2019). This finding indicates that rubber yield and residues are important sources of soil C and N inputs (Agovino et al., 2019).

The results also indicate that non-significant differences in soil C and N storage may confirm the input and output balance of carbon and nitrogen. However, further research is required to validate this perspective (Dourado and De Moraes, 2018). For example, the contribution of rubber tree root systems to soil C and N storage is essentially unclear. Moreover, caution must be exercised while interpreting and applying this conclusion on a broader scale as it requires long-term observation (Wang et al., 2022). The findings show that the total P content and available P content in the soil significantly decreased during the rainy season. Specifically, the total P content in the rainy season decreased by 51.0% compared with the dry season, while the available P content in the soil decreased by 84.8% compared with the dry season (**Figure 4**). Additionally, the rainy season has a significant impact on the total potassium content and available potassium content of the soil. The total K content in the rainy season soil decreased by 21.1% compared with the dry season, and the available K content in the soil decreased by 51.2% compared with the dry season.

Several mechanisms can explain the significant decreases in soil total P, available P, total K, and available K during the rainy season (**Figure 4**, **Table 2**), which we summarize from three aspects: rubber tree absorption, runoff leaching, and latex removal (Hu et al., 2016; Yang X. et al., 2019). First, enhanced soil moisture and temperature during the rainy season stimulate root activity and nutrient uptake, with rubber trees having a dense shallow root system (0–30 cm) that accelerates absorption of available P and K to support rapid growth and latex synthesis (Qi et al., 2024). Second, the rainy season receives approximately 80% of annual precipitation, and the weak canopy interception capacity of monoculture rubber plantations leads to substantial surface runoff that carries away fine soil particles enriched with P and K, while intense rainfall also promotes leaching of dissolved nutrients from the topsoil. Third, rubber tapping removes not only latex—which contains K as a major cation for osmotic balance—but also small amounts of P associated with phospholipids and ATP; during the rainy season, more frequent tapping and higher latex yield result in greater nutrient removal (Liu et al., 2018). Together, these three mechanisms explain the significant seasonal decline in soil P and K, despite the concurrent numerical increase in rubber yield, which is primarily limited by water and temperature rather than by P/K availability in this system (Liu et al., 2025).

### 3.3 Relations between rubber yield, yield stability, and soil C and N content

**Figure 5:**
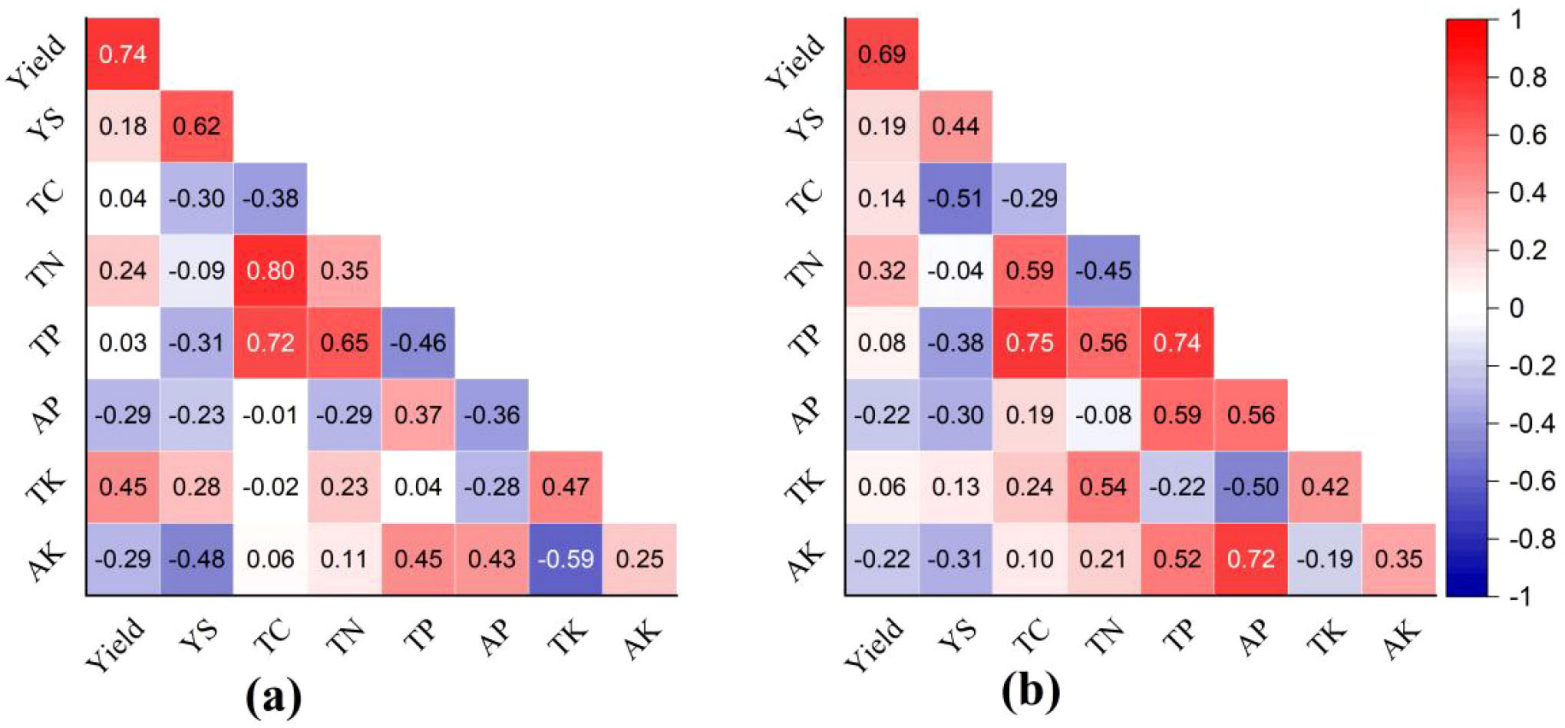
Correlation matrix of rubber yield, yield stability (YS), soil total carbon (TC), total nitrogen (TN), total phosphorus (TP), available phosphorus (AP), total potassium (TK), and available potassium (AK) at 0–40 cm soil depths during **(a)** dry (November–April) and **(b)** rainy (May–October) seasons in 2018. Correlation coefficients are color-coded (blue: positive; red: negative) with numerical values displayed. Hierarchical clustering was performed using Euclidean distance and Ward’s linkage method to group variables with similar correlation patterns.

The results showed that there was no evident relationship between rubber yield and changes in soil C and N content in the rainy and dry seasons. Additionally, the decline in soil P and K contents did not show a significant negative impact on rubber yield (**Figure 5**). Rubber tapping may reduce carbon input from plant sources, so a reduction in soil C content could be hypothesized based on this negative correlation (between tapping and plant-derived C inputs) (King and Blesh, 2018). In contrast, rubber tapping and reduced fresh and dead litter inputs may protect soil C content because there is no associated priming effect (Wang et al., 2019).

Additionally, the seasonal input of rubber tree litter (such as leaves and branches) may be relatively stable. Although quantity of litter is slightly higher in rainy season, decomposition is accelerated by high temperature and humidity. In the dry season, litter amount may decrease but the decomposition rate is also reduced. Thus, the overall carbon and nitrogen mineralization and fixation tend to balance out (Nottingham et al., 2015). Although the net effect of rubber tapping on soil C content remains inconclusive, this can explain the lack of correlation between rubber yield and soil C and N contents.

Rubber trees have a unique nutrient demand pattern due to continuous latex removal. Latex contains approximately 0.2–0.4% potassium (K) on a fresh weight basis, which plays a critical role in maintaining osmotic potential in laticifers and stabilizing rubber particles (Liu et al., 2025). Phosphorus (P) is required for ATP synthesis during rubber biosynthesis. Unlike annual crops that remobilize nutrients from senescing tissues before harvest, rubber tapping removes nutrients directly from the phloem year-round, creating a sustained nutrient export that may decouple surface soil nutrient status from aboveground productivity.

Soil K and P contents decreased significantly in the rainy season compared to the dry season (**Figure 4**). No clear temporal relationship was observed between these nutrient changes and seasonal rubber production over the study period (**Figure 5**). In view of the decline in soil K and P contents in rainy season, the nutrient reabsorption rate of rubber tree increases before old leaves senesce and abscise, which realizes the optimization of internal circulation, and alleviates the impact of soil available P and available K on latex synthesis (Le Guen et al., 2011). Additionally, although the contents of total P and K in soil decreased, seasonal fertilization in rubber plantation management can quickly replenish these soil nutrient contents and maintain the available phosphorus and potassium concentrations in the rhizosphere to support the growth of rubber (Medlyn et al., 2011). Additionally, dynamic monitoring of the soil solution showed that the concentration of available potassium could be temporarily restored to above the critical value after fertilization. The interaction between yield stability and soil C and N content has been studied for many years, but no consensus has still been reached (Chen et al., 2020, Chen et al., 2022a; Ray et al., 2015). It is speculated that high temperatures and abundant precipitation can compensate for nutrient deficiencies and thus facilitate the maintenance of yield and yield stability despite decreased soil K and P contents. The study found that there was no clear relationship between the yield stability and the changes of soil C and N content after 5 years, mainly because the observation period was not long enough, and making it impossible to capture the non-linear responses of yield stability to nutrient depletion.

### 3.4 Uncertainties and implications for long-term studies

A key physiological feature of rubber trees relevant to this study is their seasonal defoliation and refoliation cycle. In Xishuangbanna, rubber trees typically shed leaves during the dry season and remain dormant, then refoliate rapidly with the onset of the rainy season. Latex tapping is suspended during the defoliation period to protect tree health (Xiao et al., 2014). This phenological pattern means that the rainy season coincides with the period of active growth and latex flow, while the dry season corresponds to physiological dormancy. Therefore, the observed seasonal differences in yield are not merely responses to contemporaneous climate but are shaped by this evolved phenological adaptation.

In different precipitation seasons, rubber yield and yield stability increased, while soil C and N contents did not show significant changes (Stewart et al., 2015; Gaudin et al., 2015). This indicates that under the investigated conditions, there may be new management strategies for fertilizer input in rubber forests. Furthermore, it has been reported that changes in rubber yield, yield stability, and soil C and N contents are related to soil physical properties (Grover et al., 2009; Raseduzzaman and Jensen, 2017) and harvest management (Dias et al., 2015; Valdez et al., 2017). The results highlight the need for more research on the environmental impact of this system before expanding it to larger areas. For example, research should be conducted on water retention and mulching treatment for rubber forests during rainy and dry seasons as potential method to optimize fertilizer supply.

A limitation of this study is that soil properties were measured only once (2018) after five years of yield monitoring; therefore, interannual trends in soil C, N, P, K could not be assessed. It should be noted that these results, derived from our 5-year study, reflect short-term effects compared with those from longer-term studies. Therefore, caution should be exercised when comparing the results with other long-term studies. Although the results of short-term and long-term studies are sometimes statistically significant, the results of short-term studies may not be consistent with long-term reality and may even form sharp contrasts with long-term reality. Therefore, to better leverage the potential of rubber planting in future climate-smart and sustainable agriculture, further analysis of the individual or combined effects of rubber forest management is needed in future research (Kuebbing et al., 2018).

To justify the recommendation for longer-term (≥10 years) continuous observations, we conducted a *post-hoc* power analysis based on the interannual variance of rubber yield observed in this study. Using the standard deviation of annual yield across the five years (SD = 0.23) and setting α = 0.05 with 80% power, we calculated that detecting a moderate effect size (Cohen’s d = 0.5) for seasonal differences would require approximately 10–12 years of monitoring. This calculation follows standard procedures for ecological field studies (White, 2018; Cusser et al., 2020). Furthermore, detecting significant changes in soil C and N stocks—which typically accumulate or deplete slowly in tropical soils—often demands decadal time scales due to high spatial variability and buffering capacity (Schmidt et al., 2011; West and Post, 2002). Therefore, the ≥ 10-year recommendation is not arbitrary but grounded in both statistical power considerations and the known dynamics of soil organic matter in tropical agroecosystems.

## 4 Conclusion

The results indicate that relative to the dry season, the rainy season can significantly increase rubber yield and yield stability. There is no significant difference in soil C and N storage during the rainy and dry seasons; however, the contents of total P, available P, total K, and available K in the soil decrease significantly during the rainy season compared with the dry season. Soil C and N storage are not correlated with rubber yield and yield stability, while the decrease in phosphorus and potassium does not show a significant impact on the improvement of rubber yield and yield stability. Nevertheless, the storage of soil C and N in rubber plantation ecosystems provides unique and valuable information for future climate-smart and sustainable agricultural development. However, continuous field observations of rubber production and soil physicochemical properties and nutrient storage in the same agricultural ecosystem during different precipitation seasons are necessary to further empirically understand the long-term flow of C, N, and other nutrients (e.g., P and K). Although long-term, large-scale research is still required, the results emphasize the necessity and value of systematic studies on rubber yield, yield stability, and soil C and N contents across rainy and dry seasons.

## Data Availability

The original contributions presented in the study are included in the article/**Supplementary Material**. Further inquiries can be directed to the corresponding authors.
