# Supplementary material for: Enhanced rubber yield and its stability during the rainy season: insights from five-year yield monitoring in Southwestern China
Source: Front Plant Sci. 2026 May 1;17:1824436. doi: 10.3389/fpls.2026.1824436 (PMC13178699; doi:10.3389/fpls.2026.1824436)
Supplement: Supplementary file 1 [file SupplementaryFile1.doc]

***Supplementary Material***

1. **Supplementary Figures**


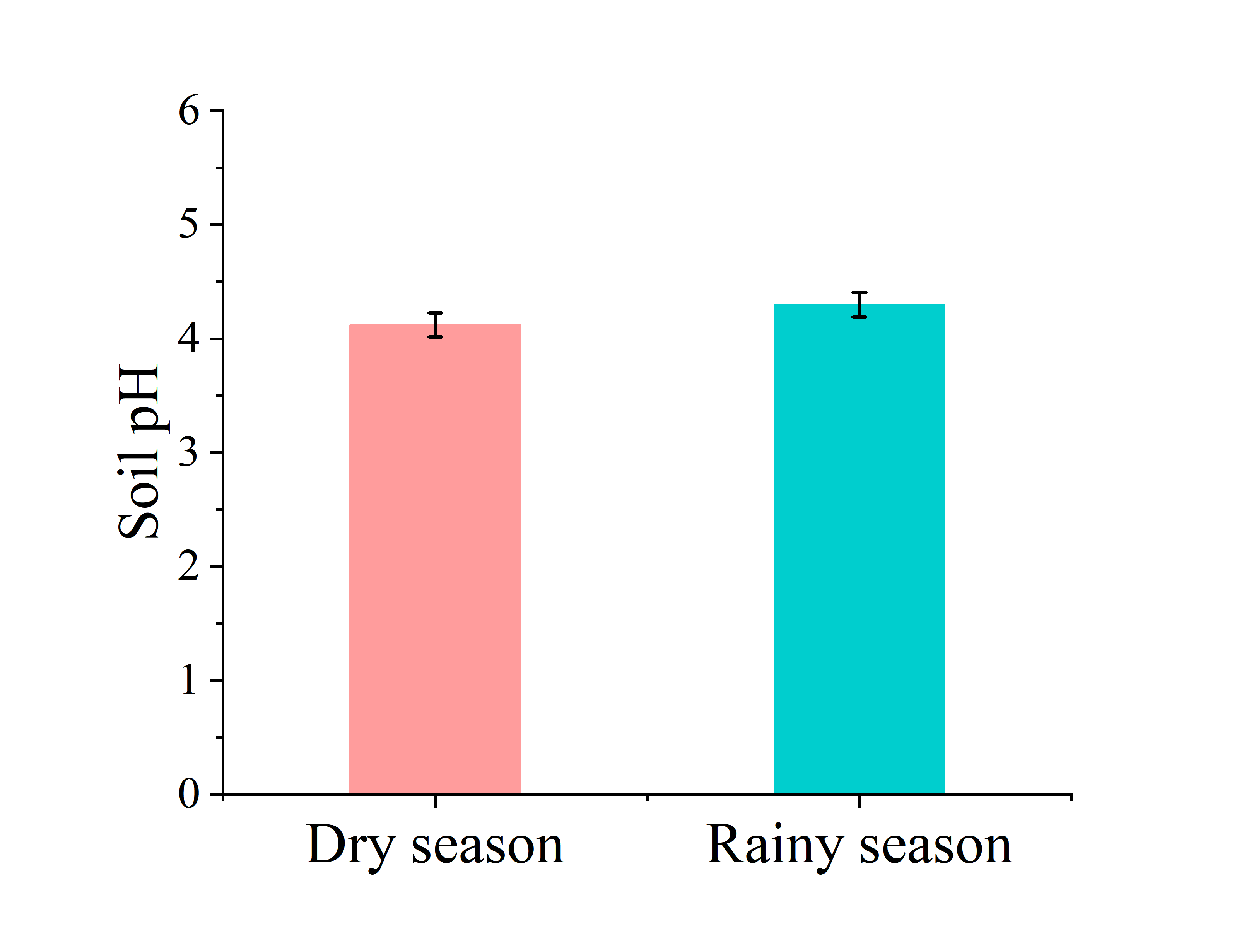


**Supplementary Figure 1.** Soil pH value in the soil from the surface to a depth of 40 cm in different seasons (rainy and dry) in 2018. Values represent mean ±standard errors for three replicates (*n* = 3).

**
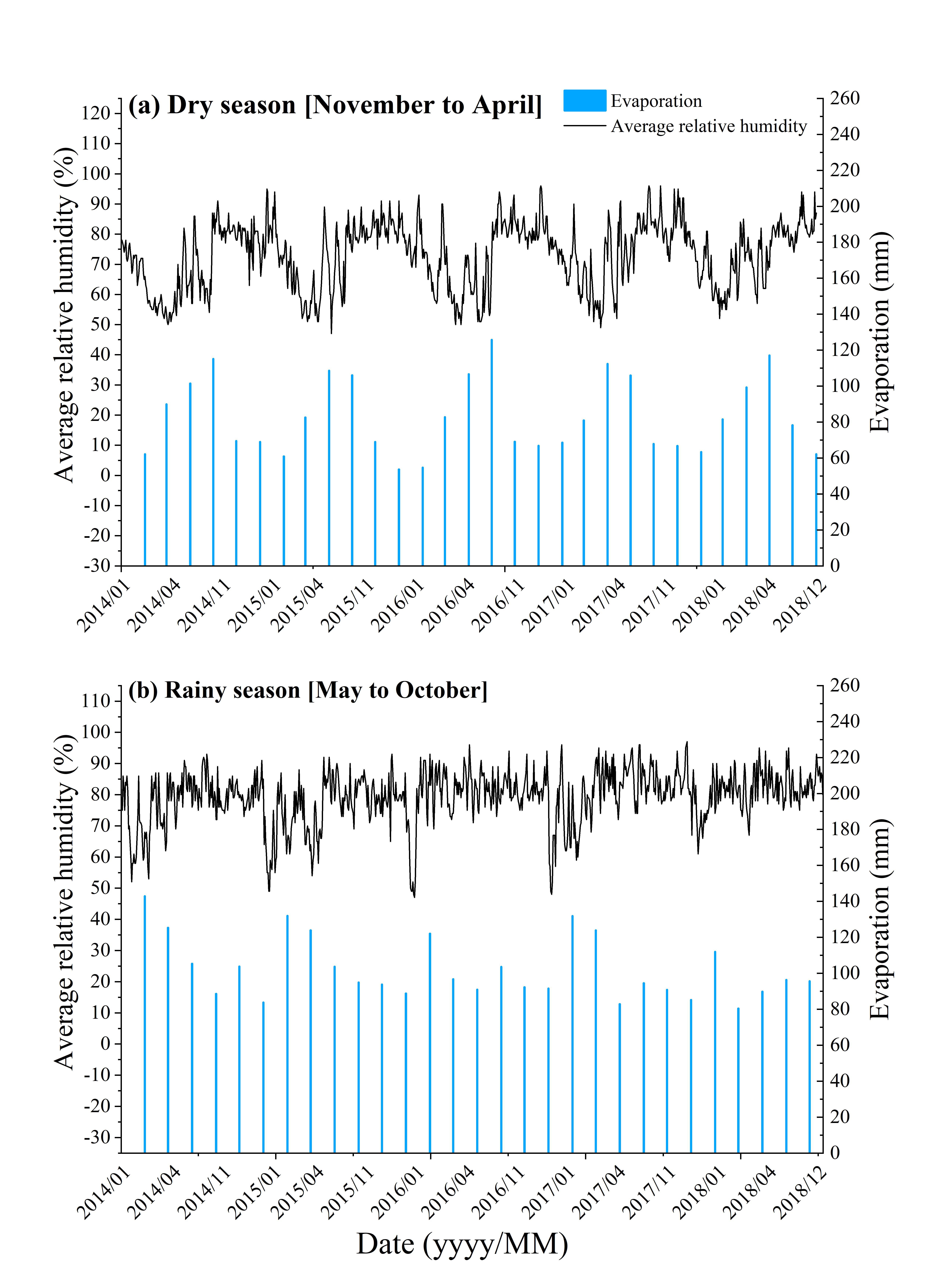
**

**Supplementary Figure 2.** Evaporation (blue bars) and average relative humidity (black curves) during (a) dry [November to April] and (b) rainy [May to October] seasons in Jinghong, Yunnan, China.

**
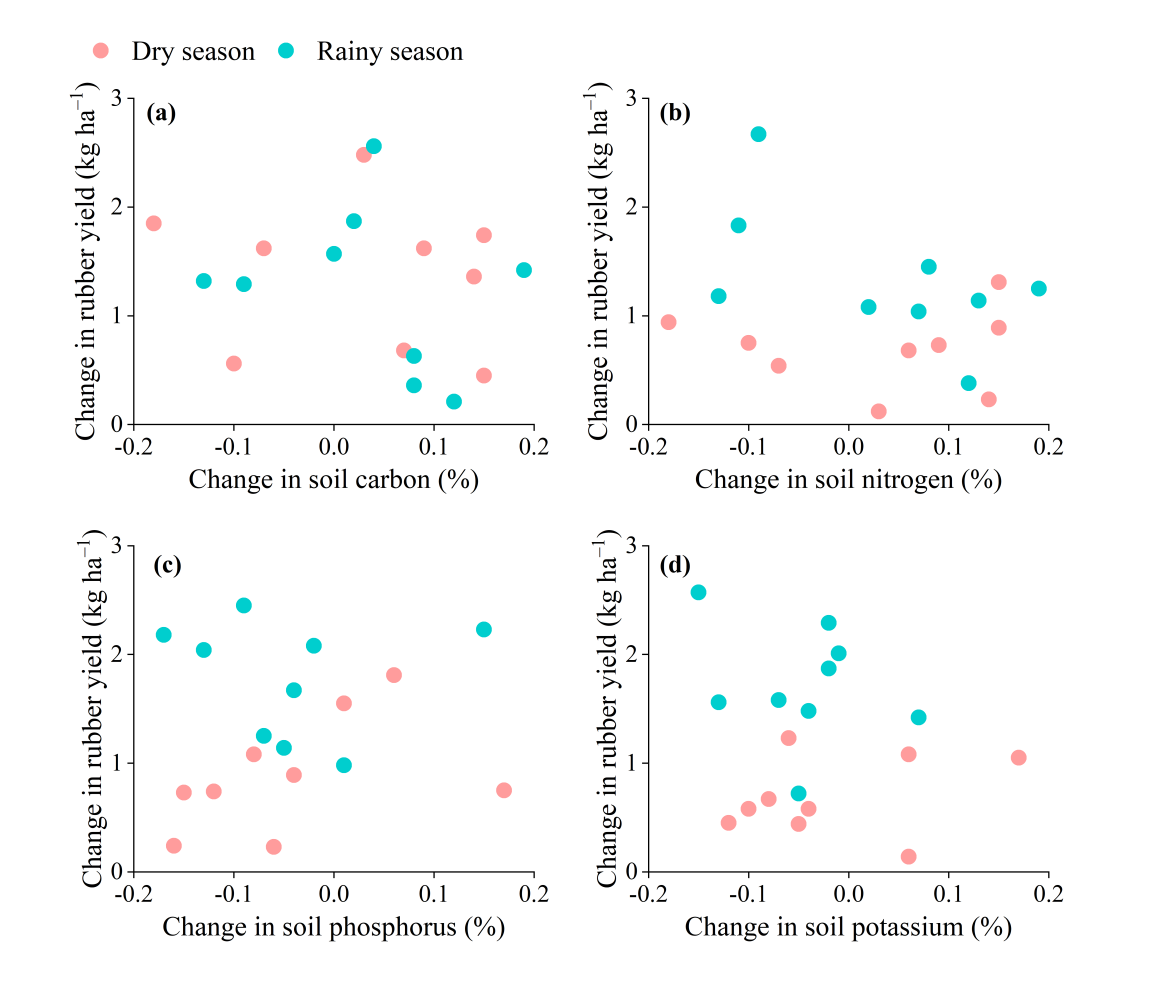
**

**Supplementary Figure 3.** Relationships across precipitation seasons between changes in rubber yield and changes in (a) soil carbon content (SOC), (b) soil nitrogen content, (c) soil phosphorus content and (d) soil potassium content from multiple soil layers. No clear relationship was observed across the dry and rainy season.
